# Structural basis for chemokine receptor CCR6 activation by the endogenous protein ligand CCL20

**DOI:** 10.1038/s41467-020-16820-6

**Published:** 2020-06-15

**Authors:** David Jonathan Wasilko, Zachary Lee Johnson, Mark Ammirati, Ye Che, Matthew C. Griffor, Seungil Han, Huixian Wu

**Affiliations:** Discovery Sciences, Medicine Design, Pfizer Worldwide Research and Development, Groton, CT 06340 USA

**Keywords:** G protein-coupled receptors, Cryoelectron microscopy

## Abstract

Chemokines are important protein-signaling molecules that regulate various immune responses by activating chemokine receptors which belong to the G protein-coupled receptor (GPCR) superfamily. Despite the substantial progression of our structural understanding of GPCR activation by small molecule and peptide agonists, the molecular mechanism of GPCR activation by protein agonists remains unclear. Here, we present a 3.3-Å cryo-electron microscopy structure of the human chemokine receptor CCR6 bound to its endogenous ligand CCL20 and an engineered Go. CCL20 binds in a shallow extracellular pocket, making limited contact with the core 7-transmembrane (TM) bundle. The structure suggests that this mode of binding induces allosterically a rearrangement of a noncanonical toggle switch and the opening of the intracellular crevice for G protein coupling. Our results demonstrate that GPCR activation by a protein agonist does not always require substantial interactions between ligand and the 7TM core region.

## Abstract (web summary)

Chemokine receptors are GPCRs involved in immune responses and regulated by small protein ligands known as chemokines. A structural study of the human CCR6/CCL20–Go complex reveals that CCL20 binds in a shallow extracellular pocket, and suggests that activation of CCR6 by CCL20 binding involves an allosteric effect on a noncanonical toggle switch.

## Introduction

Chemokine receptors are GPCRs regulated by small protein ligands known as chemokines, which are 8–10 kDa proteins with a globular core structure stabilized by 1–2 conserved disulfide bridges^1^. According to the spacing of the conserved N-terminal cysteine residues, chemokines are structurally grouped into the CC, CXC, CX_3_C, and C chemokines^2^. Through chemokine receptors, chemokines can regulate leukocyte migration, activation, differentiation, and survival during the immune response, and therefore are involved in numerous diseases including inflammation, cancer, and autoimmune disorders^2^. To date, three chemokine–receptor complex crystal structures have been reported^3–5^. However, all chemokine ligands in the crystal structures are either an antagonist or inverse agonists to the co-crystallized receptors. On the other hand, the structural elucidation of agonist-bound GPCRs coupled to G proteins has provided a tremendous amount of insight into GPCR activation by small molecule or peptide ligands^6^. Nonetheless, the molecular basis of GPCR activation by protein agonists remains elusive. Thus, structural elucidation of a cognate chemokine–chemokine receptor complex is not only important for understanding the molecular basis of chemokine receptor activation by native agonists, but it could also expand our knowledge of the mechanism of GPCR activation by ligands of a distinct chemical nature.

CCR6 is a CC chemokine receptor that is selectively expressed in immature dendritic cells and memory T-cells^1^. It primarily couples through G_i/o_ proteins upon activation. CCR6 plays an important role on skin and mucosal surfaces under homeostatic and inflammatory conditions, particularly through the recruitment of proinflammatory IL17-producing T-cells to sites of inflammation. Therefore, CCR6 is a potential target for several inflammatory and autoimmune diseases including psoriasis^7^ and inflammatory bowel disease^8^. In human and mouse, CCR6 is exclusively activated by the chemokine CCL20, which is known to interact only with CCR6^7^. Intrigued by the unique exclusivity of this chemokine–chemokine receptor pair, we sought to understand the molecular basis of the ligand recognition specificity exhibited by this receptor.

To this end, we solved a cryo-EM structure of the human CCR6 in complex with CCL20 and a Go protein. Our structure reveals several interactions that are likely to be important for CCL20-mediated CCR6 activation, supported by previous mutagenesis studies and molecular dynamics simulations. Notably, CCL20 binds to a shallow extracellular pocket of CCR6, in contrast to the deep agonist-binding sites observed in other class A GPCRs. Thus, we propose that CCL20 activates CCR6 by a unique allosteric mechanism.

## Results

### Forming a stable CCR6/CCL20–Go complex

**Fig. 1:**
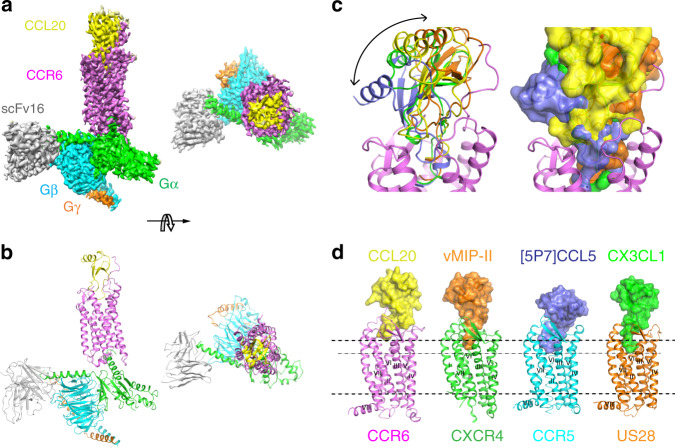
Cryo-EM structure of the CCR6/CCL20–Go–scFv16 complex and comparison with other chemokine–GPCR complexes. **a** Orthogonal and extracellular views of the cryo-EM map of the CCR6/CCL20–Go heterotrimer complex colored by subunit. CCR6, light magenta; CCL20, yellow; Gαo Ras-like domain, green; Gβ, cyan; Gγ, orange; scFv16, gray. **b** Model of the CCR6/CCL20–Go complex in the same views and color scheme as shown in (**a**). **c** Overlay with other known inactive chemokine–chemokine receptor complex structures reveals massive diversity in chemokine binding mode (CCR5/[5P7]CCL5, CXCR4/vMIP-II, US28/CX3CL). **d** Comparison of the depth into which each chemokine N terminus reaches in the orthosteric pocket of its cognate receptor. CCL20 binds in a shallow pocket at the extracellular surface with minimal contact to the 7TM core. Heavy dotted lines highlight the membrane boundaries, and light dotted line is the deepest chemokine binding position revealed by US28/CX3CL1 complex structure.

Successful expression and purification of the human wild-type CCR6 was enabled by fusing a thermostabilized apocytochrome b562 (BRIL)^9^ to the N terminus of the receptor which comprises 38 amino acid residues before TM1 (see Supplementary Information, Sequences used in this study). The CCL20 was expressed as an N-terminal calmodulin fusion chimera. Upon TEV-mediated proteolytic cleavage at a modified sequence of ENLYFQ/A, a wild-type CCL20 protein which preserves the native N-terminal alanine residue was obtained (see Supplementary Information, Sequences used in this study). To form a stable heterotrimeric G protein complex, we employed an engineered Go, miniGo which has proven improved stability^10^. We also introduced the reported single-chain variable fragment (scFv16)^11^ in the complex formation to slow down the dissociation of the final nucleotide-free CCR6/CCL20–Go complex. The complex was formed by incubating all the protein components in the presence of apyrase to remove residual GDP. Subsequently, the complex was subjected to affinity purification using a FLAG-tag engineered to the N terminus of CCR6 (Supplementary Fig. 1). The purified complex was vitrified and imaged using a Titan Krios microscope (FEI) equipped with a K2 Summit direct-electron detector and energy filter (Gatan Quantum LS). Following imaging and data processing, a cryo-EM reconstruction with a resolution of 3.3 Å was obtained (Fig. 1, Supplementary Figs. 2, 3 and Supplementary Table 1), which exhibited well-resolved side-chain density particularly around CCR6, allowing confident model building from Y27 to C336 of the GPCR. Robust density was also observed for the Go complex as well as the scFv16, whereas the density map in the region of the chemokine CCL20 was noisier, indicating a dynamic interaction between the native chemokine and its receptor (Supplementary Fig. 2c, e).

### Overall structure of the CCR6/CCL20 complex

The cryo-EM map reveals that CCR6 possesses a canonical GPCR topology, consisting of seven transmembrane (7TM) α-helices, three extracellular loops (ECLs), three intracellular loops (ICLs), and a short helix (helix 8) that runs parallel to the membrane (Fig. 1a, b). ECL2 adopts a β-hairpin structure, similar to those seen in other class A γ-subfamily receptors^3–5,12^. CCL20 binds to the endogenous pocket located at the extracellular side of CCR6, making substantial interactions with all three ECLs. In addition, the N-terminal residues Y27 to L38 from CCR6 wrap onto the globular core of CCL20, serving as another critical docking site for chemokine binding (Fig. 1b). There is no density for residues preceding Y27, indicating the mobile nature of these residues and their lack of ligand interaction. Likewise, density for the fusion protein BRIL is also missing from the map. The N terminus of CCR6 is stapled to ECL3 via a highly conserved disulfide bond (C36^Nt^-C288^ECL3^), which provides additional binding surface for CCL20. Supporting this structural observation, disruption of this disulfide by mutating either C36 or C288 has been shown to impair CCR6 response to CCL20 stimulation^13^. The N terminus of CCL20 binds in the extracellular crevice of the 7TM core of CCR6, making major interactions with the receptor’s ECL2 and N terminus. Similar to the previously solved chemokine antagonist-bound GPCR structures CXCR4/vMIP-II, US28/CX3CL1, and CCR5/[5P7]CCL5^3–5^, the CCR6/CCL20 complex reveals a highly extensive binding interface which is not spatially isolated, different than that predicted by the two-site paradigm where two spatially separated contact sites are proposed to mediate chemokine–chemokine receptor interactions^4,14,15^.

Previously solved structures exhibited substantial variation in the binding poses of the chemokine antagonist/inverse agonists to the receptors. The binding mode of the agonist CCL20 to CCR6 most resembles those observed for the CXCR4/vMIP-II^4^ and US28/CX3CL^3^ complexes (Fig. 1c). However, differences are observed in the depth into which the chemokine N terminus binds in the orthosteric pocket of the receptor, as the antagonist/inverse agonists reach 4–7 Å deeper into their respective receptors than CCL20 does in CCR6 (Fig. 1d). This is consistent with the observation that CCL20 has the shortest N terminus among all chemokines^5^ (Supplementary Fig. 4).

### The N terminus of CCL20 binds to a shallow pocket

**Fig. 2:**
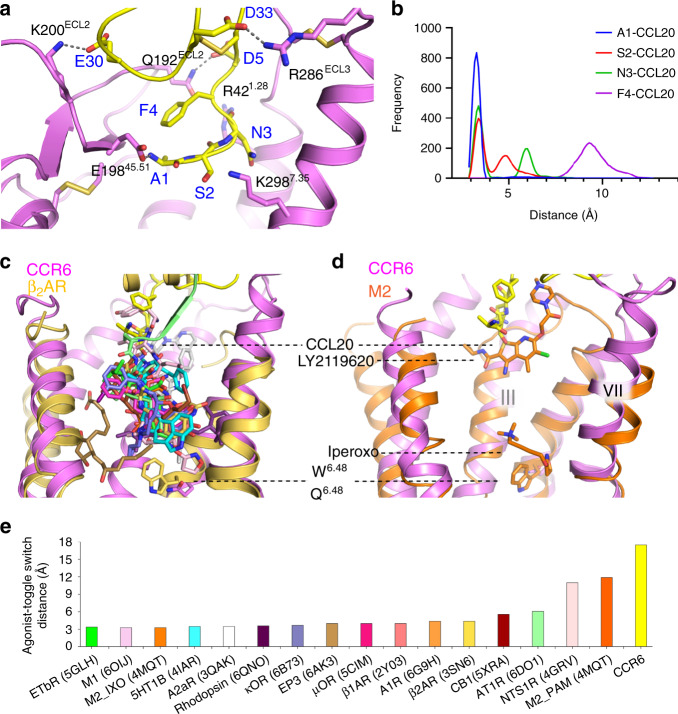
The N terminus of CCL20 binds in a shallow pocket in CCR6 close to the extracellular surface. **a** N terminus of CCL20 binding in the orthosteric pocket of CCR6 with critical polar interactions shown by dotted lines. **b** Frequency distribution of the distance between CCR6 E198^45.51^ and CCL20 N-terminal NH_3_^+^ during MD simulations using the indicated CCL20 N-terminal truncations. **c** Comparison of agonist-binding positions between other class A GPCRs and CCR6. CCL20 is colored in yellow. Agonist-bound GPCRs used in the comparison are: 5HT1B (4IAR, cyan), A1R (6D9H, orange), A2aR (3QAK, white), AT1R (6DO1, lime), β1AR (2Y03, deep salmon), β2AR (3SN6, yellow orange), CB1 (5XRA, brown), ETbR (5GLH, green), EP3 (6AK3, sand), κOR (6B73, slate), M1 (6OIJ, light violet), μOR (5C1M, magenta), NTS1R (4GRV, light pink), and Rhodopsin (6QNO, purple). Side-chains of Q^6.48^ of CCR6 and W^6.48^ of β2AR are shown in stick representation for comparison. Parts of TM3 and TM4 have been removed for clarity. **d** Comparison of CCR6 and PAM-agonist bound M2 (4MQT, orange). The CCL20 N-terminal binding site resembles the PAM LY2119620 binding site in M2. Parts of TM3 and TM4 have been removed for clarity. **e** Summary of the distance between the agonist-binding site and the toggle switch in different GPCRs.

The N terminus of CCL20 is comprised of five amino acids, NH_2_-ASNFD, which adopt an extended structure in the complex (Fig. 2a). D5 of CCL20, the last N-terminal residue before the CC motif, points toward Q192^ECL2^ and R42^1.28^ (superscripts indicate Ballesteros–Weinstein numbering for GPCRs^16^) of CCR6, interacting with these two side-chains via a hydrogen bond and a salt bridge, respectively. These polar interactions likely direct binding of the N terminus of CCL20 to the 7TM core of CCR6 and therefore could be crucial for CCR6 activation^15^. In support of this, a dramatic decrease in chemotactic potency was observed for CCL20 variants bearing D5A or D5K mutations^15^. The extreme N terminus of CCL20 (NH_2_–AS) binds in close proximity to E198^45.51^ and K298^7.35^, both of which are CCR6-specific side-chains (Supplementary Fig. 5). The α-amino group of the N-terminal alanine is likely protonated at physiological pH^17^, which may allow a salt bridge to form with the carboxylate of E198^45.51^. Aside from D5, we did not observe notable side-chain-mediated interactions between residues 1–4 of CCL20 and CCR6. This finding could explain the observed tolerance of various N-terminal point mutations at CCL20 with respect to its binding affinity and activity^15^.

The N-terminal length of various chemokines has been shown to be critical for receptor activation^2^. However, CCL20 chemotactic potency has demonstrated good tolerance toward N-terminal truncations^15^. There are two naturally occurring CCL20 variants in humans, CCL20 (1–70) and CCL20 (2–70), both of which exhibited similar potency toward CCR6^18^. Furthermore, an engineered CCL20 variant starting with N3 (3–70) retained chemotactic activity, although with lower potency. Conversely, further truncations beyond the third residue converted the chemokine into a potent antagonist capable of inhibiting full-length CCL20-mediated chemotaxis^19^. The extended conformation adopted by the N terminus of CCL20 in the complex suggests that the α-amino group of the N-terminal S2 or N3 in the truncated isoforms may also be able to reach down to E198^45.51^ and form the same salt bridge as full-length CCL20, whereas truncations beyond N3 would prevent the N-terminal residue from reaching this site, converting the chemokine into an antagonist. To test this hypothesis, we performed molecular dynamics simulations of the binding of four CCL20 N-terminal truncation variants to CCR6. In a simulation of over 240 nanoseconds, the N terminus of CCL20 (1–70) remained close to E198^45.51^, within a distance that facilitated stable salt bridge formation (Fig. 2b). Similar salt bridges were also observed for CCL20 (2–70) and CCL20 (3–70), although with reduced frequency. Notably, the salt bridge interaction between the chemokine N terminus and E198^45.51^ was totally abolished in the simulation with CCL20 (4–70)–CCR6 complex. Instead, a new salt bridge was observed between the side-chains of E198^45.51^ and K298^7.35^ in CCR6 (Supplementary Fig. 6), likely representing an inactive state of the receptor. Taken together, this structural observation implies that the salt bridge between the CCL20 N terminus and E198^45.51^ of CCR6 could play a critical role in the receptor activation process.

The shallow binding mode of CCL20 reveals an unexpected activation mechanism of CCR6 among class A GPCRs. The agonist-bound class A GPCR structures solved to date report deep agonist-binding sites that approach the toggle switch position 6.48 (Fig. 2c, d). Such a binding mode has been proposed to cause a structural rearrangement at the toggle switch, leading to receptor activation^11,20–22^. On the contrary, the N terminus of CCL20 does not bind near the toggle switch (Fig. 2e), but rather binds close to the positive allosteric modulator (PAM) binding site of the M2 receptor (Fig. 2d, e)^23^. This observation implies that neither extensive interactions with the 7TM core nor direct contact with the toggle switch residue are necessary for activation of this class A GPCR, demonstrating the diverse activation mechanisms of this receptor class.

### Structural features of CCR6 activation by CCL20

**Fig. 3:**
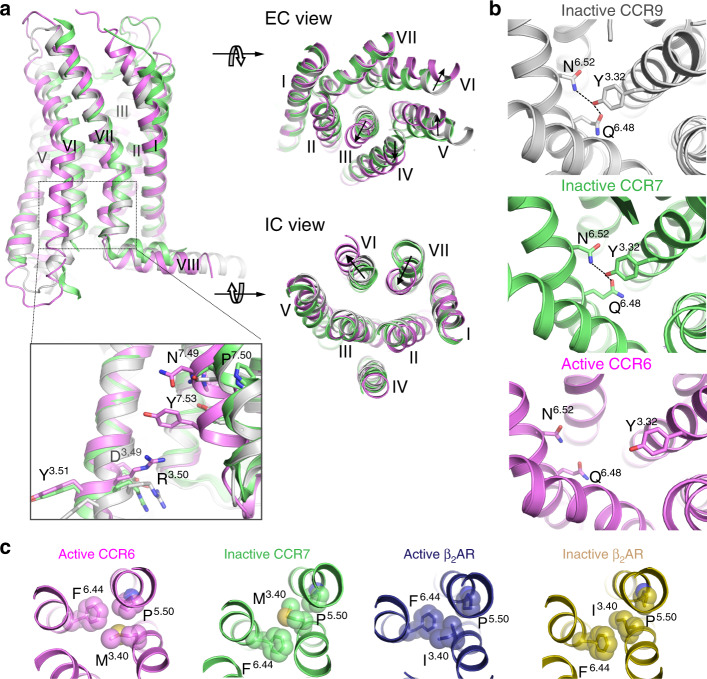
CCL20-bound CCR6 is in the active state. **a** Structural superposition of CCR6 7TM (light magenta) with CCR7 (green) and CCR9 (white) from the orthogonal, extracellular, and intracellular views. Conformations of the D^3.49^R^3.50^Y^3.51^ and N^7.49^P^7.50^xxY^7.53^ motifs in the active CCR6 and the inactive CCR7 and CCR9 structures are highlighted in the insert. Parts of TM5 and TM6 have been removed for clarity. **b** Conformation of the conserved side-chains Y^3.32^, N^6.52^ and Q^6.48^ of CCR9, CCR7, and CCR6. **c** Conformation of the P^5.50^M^3.40^F^6.44^ motif in inactive CCR7 (green) and active CCR6 (light magenta), and comparison with the conformation of the P^5.50^I^3.40^F^6.44^ motif in the inactive (2RH1, yellow orange) and active β_2_AR (3SN6, deep blue).

Next, we examined the molecular features of CCR6 activation by CCL20. Superposition of the active CCR6 structure with the inactive structures of the closely related CC chemokine receptors CCR7^24^ and CCR9^25^, neither of which has ligand bound in the orthosteric pocket, reveals significant structural changes of the extracellular pocket in CCR6 upon agonist binding. These changes include outward movements of TM3, TM4, and TM6 and an inward movement of TM5 (Fig. 3a). Interestingly, all three receptors contain a glutamine residue at the toggle switch 6.48 position (Q267^6.48^ in CCR6) and several other conserved residues nearby, including Y125^3.32^ and N271^6.52^. In both the CCR7 and CCR9 inactive-state structures, hydrogen-bonding interactions are observed between Y^3.32^, N^6.52^ and Q^6.48^, which could stabilize TM3 and TM6 in a close-contact conformation (Fig. 3b). In the active CCR6 structure, interactions between these three side-chains are no longer observed, which is further associated with the outward movement of TM3 and TM6 on the extracellular side. Such a rearrangement of side-chain interactions around the toggle switch position may mediate the receptor activation process in response to chemokine binding at the extracellular side of CCR6.

P^5.50^I^3.40^F^6.44^ is another important motif that undergoes significant conformational changes upon receptor activation in class A GPCRs^26^. In CCR6 and CCR7, this motif is replaced by P226^5.50^M133^3.40^F263^6.44^. A comparison of the active CCR6 and inactive CCR7 structures reveals a rotation of F^6.44^ toward P^5.50^ as well as a movement of the M^3.40^ side-chain toward TM6 in the active CCR6 structure, consistent with the previously known structural rearrangement of this motif during receptor activation^26,27^ (Fig. 3c). Close to the intracellular crevice, CCR6 harbors the canonical fingerprint motifs of class A GPCRs, D142^3.49^R143^3.50^Y144^3.51^, and N312^7.49^P313^7.50^xxY316^7.53^ (Fig. 3a, inset)^20,26^. Both motifs are in active conformations similar to other published active-state GPCR structures^11,26,28^ (Supplementary Fig. 7). Furthermore, when compared with the inactive CCR7 and CCR9 structures, CCR6 undergoes a 6.4-Å outward movement of TM6 and a 4.6-Å inward movement of TM7 at the intracellular tips, which opens the intracellular crevice to allow G protein coupling (Fig. 3a).

### Structure of the CCR6–Go interface

**Fig. 4:**
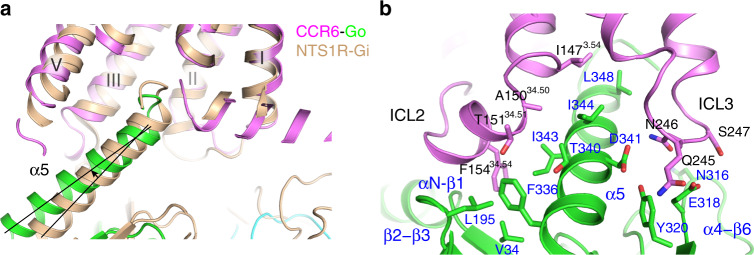
Interactions between CCR6 and Go. **a** Comparison of the relative orientation of Go α5 (green) binding to CCR6 (light magenta), and Gi (wheat) binding to NTS1R (wheat). **b** Interactions between α5 of Go with ICL2 and ICL3 of CCR6. Residues at this interaction interface are shown in stick representation, with CCR6 side-chains labeled in black, and Go side-chains labeled in blue.

**Fig. 5:**
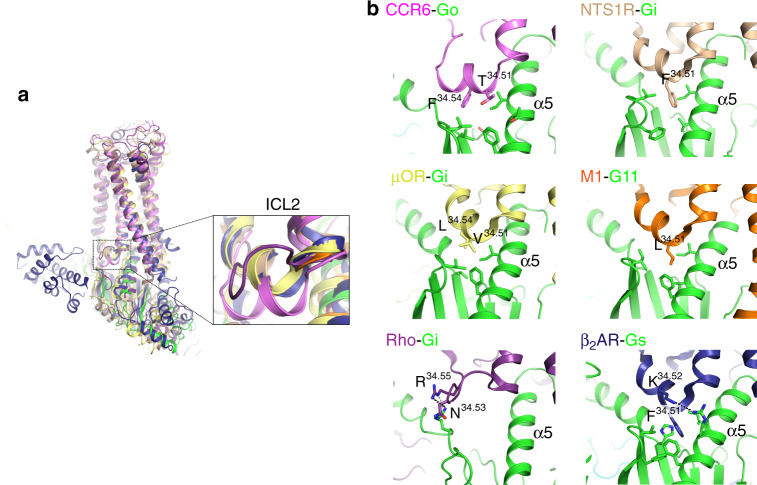
Diverse conformations adopted by ICL2 for G protein coupling. **a** Structural alignment of GPCR–G protein complexes demonstrates ICL2 of CCR6 (light magenta) adopts a unique conformation when coupled to G proteins. Structures used for comparison are: NTS1R–Gi (6OS9, wheat), μOR-Gi (6DDE, pale yellow), M1-G11 (6OIJ, orange), Rhodopsin-Gi (6QNO, purple), and β2AR-Gs (3SN6, deep blue). **b** Interactions between ICL2 and the Gα subunit in different GPCR–G protein complexes represented as in (**a**), with only receptor side-chains labeled for clarity. Gα proteins are colored in green for clarity.

Similar to other G protein complex structures, in the CCR6–Go complex the C terminus of the α5 helix adopts a loop conformation, binding to the intracellular crevice of the 7TM bundle of CCR6^11,28–30^. When compared with the recent NTS1R–Gi complex structures, which elucidated two conformational states of the G protein when coupled to the activated receptor^31^, the CCR6–Go complex closely resembles the canonical state with major contacts between α5 and the receptor coming from TM3 and ICL2 (Supplementary Fig. 8). However, the N terminus of α5 in the CCR6 complex deviates from that of the NTS1R–Gi complex by a small rotation (~7°) along the helix, orienting the N terminus of α5 closer to the intracellular surface of CCR6 (Fig. 4a). We notice deviations of the α5 docking position were also observed in other GPCR–G protein complex structures when compared with the canonical NTS1R–Gi complex structure (Fig. 5). The difference observed in CCR6 is likely attributed to the more extensive contacts made by ICL2 and ICL3 with α5, sandwiching this helix via both hydrophobic and polar interactions (Fig. 4b). ICL2 of CCR6 forms a two-turn α-helical structure with F154^34.54^ docking into a hydrophobic pocket formed by G protein side-chains from the αN-β1 loop, the β2-β3 loop, and α5. In most other receptor–G protein complexes, interactions between ICL2 and Gα are usually made by a hydrophobic side-chain from the 34.51 position to the β2-β3 loop and α5^11,27,28,31^ (Fig. 5b). Due to this unique interaction, ICL2 of CCR6 adopts a more G protein-engaging conformation compared with the ICL2 conformations found in other receptor–G protein complexes^11,28,31,32^ (Fig. 5a). The Go coupling is further stabilized by interactions between ICL3 and the α4-β6 loop (Fig. 4b). The α4-β6 loop is one of the most important receptor binding sites for coupling and specificity, although the interface from the receptor for such interactions can be quite variable^31^. Overall, the diversity and plasticity of the interaction interface between GPCRs and G proteins structurally elucidated to date reveals a molecular basis for how G proteins can couple to various receptors that have divergent intracellular surface features.

## Discussion

**Fig. 6:**
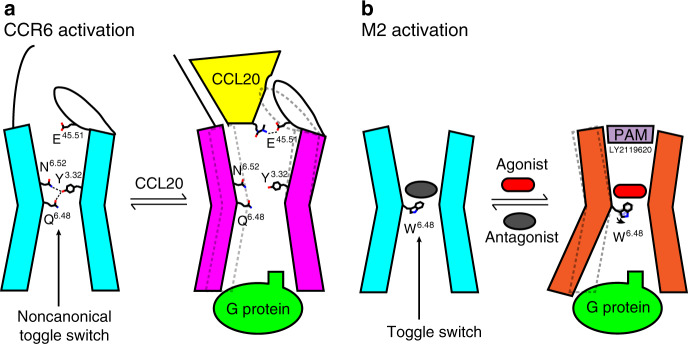
The diverse activation mechanisms of class A GPCRs. **a** Proposed model of CCR6 activation by CCL20. In the absence of CCL20, the noncanonical toggle switch Q^6.48^ is involved in an H-bond network that stabilizes CCR6 in an inactive state. Binding of CCL20 is associated with the disruption of these interactions, facilitating CCR6 activation and downstream G protein coupling. The salt bridge between the N-terminal NH_3_^+^ group of CCL20 and the CCR6-specific side-chain E^45.51^ from ECL2 is illustrated in the cartoon. **b** Activation mechanism of the M2 receptor as demonstrated by previous structural studies. The canonical toggle switch W^6.48^ adopts a different rotamer conformation when agonist is bound, which is the key trigger for M2 activation and downstream G protein coupling. The active state of M2 has a smaller extracellular pocket which is utilized by a PAM, LY2119620. The CCL20-binding site in CCR6 resembles the shallow PAM-binding site observed in M2.

The CCR6/CCL20–Go complex structure reveals a unique mechanism of GPCR activation by a protein agonist. The binding site of the CCL20 N terminus, a key trigger for CCR6 activation, is located close to the extracellular surface of the receptor and deviates from the well-studied deep agonist-binding sites embedded in the 7TM core for most class A GPCRs. Instead, it binds rather distal to the noncanonical toggle switch of CCR6, closely resembling the PAM-binding site observed in M2 receptor^23^ (Figs. 2d and 6b). Interestingly, the ionic interaction between the N-terminal amino group of CCL20 and the CCR6 side-chain E198^45.51^ as highlighted in the cartoon shown in Fig. 6a, is the only specific interaction observed between the N terminus of the chemokine and the receptor. The absence of the H-bond network between TM3 and TM6 mediated by the noncanonical toggle switch Q267^6.48^ in the active CCR6, unlike that observed in the inactive CCR7 and CCR9 structures^24,25^, likely facilitates the movement of the 7TM bundles to accommodate G protein coupling. Such an activation mechanism is reminiscent of the activation of class C GPCRs, whose endogenous agonists bind to an orthosteric site located on the N-terminal extracellular domain and make no direct contact with the 7TM helices^33,34^. It is noteworthy that this binding mode may be specific for CCR6/CCL20, as other chemokines have comparatively longer N termini that could potentially reach deeper into the 7TM bundle.

CCR6–CCL20 represents a unique pair within the chemokine receptor-chemokine family with an excellent specificity between the receptor and its cognate ligand^1,15^. It is highly possible that the unique binding mode of CCL20 and CCR6 discussed above underlies such specific recognition. CCL20 has the shortest N terminus among the chemokine family, which allowed the positioning of the N-terminal α-amino group to interact with the CCR6-specific side-chain E198^45.51^. We notice that there is a pocket in CCR6 at the typical orthosteric site of class A GPCRs, though not utilized by the wild-type CCL20, which presumably could accommodate an elongated N terminus to a certain extent. Nonetheless, increasing the N-terminal length of CCL20 could potentially hamper the formation of an effective ionic interaction between the chemokine with the side-chain E198^45.51^ of CCR6. Indeed, a CCL20 mutant bearing a penta-alanine insertion to the N terminus substantially reduced the activity of the chemokine^15^. In addition, several polar interactions are present between the globular core of CCL20 and the ECLs of CCR6, including salt bridge interactions between E30 (CCL20) and K200^ECL2^ (CCR6), and D33 (CCL20) and R286^ECL3^ (CCR6). The interaction between E30 and K200^ECL2^ is specific to the CCR6/CCL20 complex (Supplementary Figs. 4 and 5), which could also potentially contribute to the specificity of CCL20 binding to CCR6.

Overall, the CCR6/CCL20 structure provides an excellent template to understand the recognition specificity of this chemokine–chemokine receptor pair. Furthermore, the unique mode of GPCR activation in response to a protein agonist revealed by the CCR6/CCL20 structure demonstrates that conformational changes that occur during class A GPCR activation are not necessarily triggered by a substantial ligand engagement with the core 7TM bundle, highlighting the versatility of the GPCR 7TM fold to receive a broad spectrum of chemical signals.

## Methods

### Expression and purification of CCR6

Human wild-type CCR6 from S2 to M374 was cloned into a pFastBac1 vector (Invitrogen) with a haemagglutinin (HA) signal sequence followed by a N-terminal FLAG-tagged BRIL fusion and a C-terminal 10 × His-tag. The CCR6 protein was expressed in Spodoptera frugiperda (*Sf9*) insect cells (ATCC) using the Bac-to-Bac Baculovirus Expression System (Invitrogen). Sf9 cells were infected at a cell density of 4 × 10^6^ cells/ml with high titer viral stock multiplicity of infection (MOI) of 5.0. At 48 h post infection, cells were collected and resuspended in hypotonic buffer containing 10 mM HEPES, pH 7.5, 10 mM MgCl_2_, 20 mM KCl, and EDTA-free cOmplete^TM^ protease inhibitor cocktail (Roche). Cells were lysed by dounce homogenization and membranes were subjected to successive hypotonic and hypertonic membrane washes followed by ultracentrifugation. The washed membranes were resuspended in buffer containing 10 mM HEPES pH 7.5, 10 mM MgCl_2_, 20 mM KCl, and 40% glycerol and stored at −80 °C for future use. To purify CCR6, the washed membranes were thawed and resuspended in buffer containing 50 mM HEPES pH 7.5, 500 mM NaCl, and 1 mg/ml iodoacetamide (Sigma). Membranes were then solubilized by adding 1% (w/v) lauryl maltose neopentyl glycol (LMNG, Anatrace), 0.2% (w/v) cholesteryl hemisuccinate (CHS, Sigma) for 4 h at 4 °C. The sample was clarified by ultracentrifugation and the supernatant was batch bound to TALON cobalt-affinity resin (Takara) in the presence of 1 M NaCl and 5 mM imidazole overnight at 4 °C. The resin was washed with successively lower concentrations of detergent, and eluted in buffer containing 50 mM HEPES pH 7.5, 500 mM NaCl, 0.003% LMNG, 0.0006% CHS, 300 mM imidazole, and 10% glycerol. The elution fractions were concentrated and desalted on a PD MiniTrap G-25 column (GE Healthcare) equilibrated in buffer containing 50 mM HEPES pH 7.5, 500 mM NaCl, 0.003% LMNG, 0.0006% CHS, and 10% glycerol. The desalted protein was concentrated, flash-frozen in liquid nitrogen, and stored at −80 °C for future use.

### Cloning, expression, and purification of CCL20

CCL20 expression and purification was facilitated by an N-terminal calmodulin (Origene) fusion to stabilize CCL20 during bacterial expression^35^. An N-terminal 10× His-tag was added to calmodulin, and a noncanonical TEV site (ENLYFQ/A)^36^ was inserted between calmodulin and CCL20 (used to preserve the N-terminal alanine of native processed CCL20). The construct was ligated into pET29b and transformed into Origami 2 cells (Novagen). Cultures derived from single colonies were grown in Luria–Bertani medium supplemented with 50 µg/ml kanamycin and 12.5 µg/ml tetracycline to mid-log phase. Protein expression was induced with 0.5 mM isopropyl-β-D-thiogalactopyranoside for 18 h at 16 °C. Cells were resuspended in chilled lysis buffer consisting of 20 mM HEPES pH 7.5, 100 mM NaCl, and 10 mM imidazole and lysed by three passes through a microfluidizer (Microfluidics). Insoluble cellular material was pelleted by centrifugation, and the clarified supernatant was applied to a prepacked nickel-NTA column (GE Healthcare) and washed extensively with lysis buffer supplemented with 500 mM NaCl and 40 mM imidazole. CCL20 was eluted in a linear gradient of 400 mM imidazole, and elution fractions were pooled and digested with TEV protease overnight at 20 °C. After digestion, the pooled elution was dialyzed with buffer consisting of 50 mM sodium acetate pH 4.5 and 150 mM NaCl, during which a white precipitate formed. The dialyzed sample was clarified by centrifugation and applied to a HiTrap S column (GE Healthcare) equilibrated in buffer containing 20 mM sodium acetate pH 4.5 and 150 mM NaCl. The sample was washed extensively and eluted in a linear gradient of 20 mM sodium acetate pH 4.5, 1 M NaCl. The purified CCL20 was buffer exchanged into 20 mM HEPES pH 7.5, 150 mM NaCl using a 3 kDa centrifugal filter (Amicon). The protein was concentrated and stored at −80 °C for future use.

### Expression and purification of heterotrimeric miniGo and scFv16

The miniGoβ_1_γ_2_ was expressed as a heterotrimer and purified using an N-terminal 6× His-tag fused to miniGo^10^. Individual constructs for each protein were cloned into a pFastBac1 vector and viruses were prepared using the Bac-to-Bac system. ATCC Sf9 cells at a density of 4 × 10^6^ cells/ml were co-infected with the three viruses in a 2:1:1 miniGo:β1:γ2 ratio, and expressed for 48 h. Cells were lysed by dounce homogenization in a hypotonic buffer containing 10 mM HEPES, pH 7.5, 10 mM MgCl_2_, 5 mM TCEP, 50 µM GDP, and cOmplete^TM^ protease inhibitor. The membranes were pelleted by ultracentrifugation followed by resuspension in buffer containing 50 mM HEPES, pH 7.5, 150 mM NaCl, 5 mM TCEP, 5 µM GDP, 1% LMNG, 0.2% CHS, and cOmplete^TM^ protease inhibitor for solubilization. The G protein heterotrimer was purified from the soluble fraction by cobalt-affinity chromatography. The heterotrimer was further purified by size exclusion chromatography using a Superdex 200 10/300 column equilibrated in buffer containing 50 mM HEPES pH 7.5, 150 mM NaCl, 0.003% LMNG, 0.0006% CHS, 5 mM TCEP, 5 µM GDP, and 10% glycerol. The G protein-containing elutions were pooled and stored at −80 °C for future use.

The N terminus of scFv16^11,37^ was modified to include an mIgκ leader sequence to promote secretion to the media. Virus was generated using a pFastBac1 vector and the Bac-to-Bac system. At 48 h post infection, the media was collected and batch bound to cobalt-affinity resin^37^. The beads were collected in a gravity-flow column and washed with high-salt buffer consisting of 20 mM HEPES pH 7.5, 500 mM NaCl, and 20 mM imidazole, and followed by a wash with low-salt buffer consisting of 20 mM HEPES pH 7.5, 100 mM NaCl, and 20 mM imidazole. The protein was eluted in buffer containing of 20 mM HEPES pH 7.5, 100 mM NaCl, and 250 mM imidazole, and further purified by size exclusion chromatography using a Superdex 200 10/300 column equilibrated in buffer containing 20 mM HEPES pH 7.5 and 100 mM NaCl. Peak fractions were pooled, supplemented with 10% glycerol, and stored at −80 °C for future use.

### Formation of a CCR6/CCL20–miniGo–scFv16 complex

To form the complex, CCR6, G protein heterotrimer, and CCL20 were mixed in a 1:1.2:10 molar ratio and incubated at 20 °C for 2 h. Apyrase (NEB) was added to remove any residual GDP present in the sample and scFv16 was added to a 1:1.2 CCR6:scFv16 molar ratio. The complex was incubated on ice for 1 h, then batch bound to M2 anti-FLAG affinity resin (Sigma) equilibrated in 20 mM HEPES pH 7.5, 100 mM NaCl for 1 h at 4 °C. The complex slurry was applied to a gravity-flow column (Bio-Rad) and washed with buffer containing 20 mM HEPES pH 7.5, 100 mM NaCl, 0.001% LMNG/0.0002% CHS. The complex was eluted in elution buffer consisting of 20 mM HEPES pH 7.5, 100 mM NaCl, 0.001% LMNG/0.0002% CHS, 0.2 mg/ml FLAG peptide, and concentrated to 7.5 mg/ml in preparation for cryo-EM analysis.

### Cryo-EM data collection and processing for the CCR6/CCL20–miniG_o_–scFv16 complex

Immediately prior to vitrification, 3 mM fluorinated Fos-Choline-8 (Anatrace) was added to the complex. The freshly purified complex (6.8 mg/ml final concentration) was applied to freshly plasma-cleaned Quantifoil R1.2/1.3 300 mesh Au holey-carbon grids (Quantifoil) and vitrified using a Vitrobot Mark IV (FEI). The grids were imaged using a Titan Krios (FEI) equipped with a Gatan K2 Summit direct-electron detector (Gatan) and a Gatan Quantum LS Imaging Filter (GIF, Gatan) with a slit size of −20 eV. Data were collected in super-resolution mode at a magnification of ×165,000 and a pixel size of 0.435 Å. A total of 5303 movies were obtained with a defocus range of −0.6 to −2.0 μm. Each movie was recorded for a total of 8 s with 0.16 s per frame. The dose rate was 10.13 e^−^/Å^2^/s, with an accumulated dose of 81.0 electrons per Å^2^.

Movie frames were subjected to beam-induced motion correction using MotionCor2^38^, and the contrast-transfer function (CTF) for each micrograph was estimated using CTFFIND-4.1^39^. Particle selection, 2D classification, initial model generation, 3D classification, and 3D refinement were carried out in Relion3.0.5^40^. 1,000,382 particles were subjected to reference-free 2D classification and averaging. 600,456 particles from the selected 2D classes were subjected to 3D classification. One class containing 230,450 particles showed detailed features for all subunits and was subjected to masked 3D refinement. Iterative cycles of masked 3D refinement, Bayesian particle polishing, and CTF refinement were performed, yielding a final map with a global resolution of 3.3 Å. The reported resolution is based on the gold-standard Fourier shell correlation (FSC) using the 0.143 criterion. All maps were corrected for the modulation transfer function of the K2 summit direct detector and sharpened by applying a temperature factor estimated during post-processing in Relion. Local resolution was determined using ResMap^41^.

### Model building and refinement

The initial template of the CCR6 transmembrane domain was derived from the active serotonin 5-HT_1B_ receptor structure solved by cryo-EM (PDB code 6G79), and model fitting was guided by bulky amino acid residues such as Phe, Tyr, and Trp. The N terminus of CCR6 was manually built up to Y27; no density was observed N-terminal of this residue (i.e., the N-terminal BRIL fusion and S2 to Y26 of CCR6), so no model was built in this region. Coordinates of the engineered Go heterotrimeric protein from the 5-HT_1B_ receptor cryo-EM structure (PDB code 6G79) were rigid-body fit into the map using Chimera^42^. scFv16 from the cryo-EM structure of the μ-opioid receptor–Gi protein complex (PDB code 6DDE) was also fit into the map in this manner. The crystal structure of CCL20 (PDB code 1M8A) was used to rigid-body fit the chemokine into the map. After docking into the EM density map, iterative manual adjustment and real-space refinement was performed using Coot^43^. A round of global refinement and minimization in real space was carried out using *phenix.real_space_refine* in Phenix^44^, followed by restrained refinement using Refmac5^45^ in the CCP4 software suite^46^. The final model was visually inspected for fit to the map, and geometry was validated using Molprobity^47^ from the Phenix software suite. Model overfitting was evaluated through refinement against one cryo-EM half map (half map 1). FSC curves were calculated between a map generated from the model and the full map, half map 1, and the other half map not used in model refinement (half map 2). Figures were generated using Chimera 1.14^42^ and PyMol 2.2.0^48^.

### Molecular dynamics simulations

MD simulations were performed in Maestro^49^ (release 2019-1, Schrödinger) using CCR6 embedded in pre-equilibrated POPC lipid bilayers and complexed with either WT CCL20 or various N-terminal CCL20 truncations. The final system was neutral and solvated with 150 mM NaCl in the water phase. For each system, MD simulations of 240 ns duration were carried out at 300 K using Desmond^50^ (Schrödinger) under standard parameters and settings. Statistical analysis of the simulation trajectories was prepared with GraphPad Prism version 8.0.2.

### Reporting summary

Further information on research design is available in the Nature Research Reporting Summary linked to this article.

## Supplementary information

Supplementary Information

Reporting Summary

## Data Availability

The cryo-EM density map for the CCR6/CCL20–miniGo-scFv16 complex is deposited in the Electron Microscopy Data Bank (EMDB) under accession code EMD-21950. The coordinates for the model are deposited in the Worldwide Protein Data Bank (wwPDB) under accession code 6WWZ. All the other data supporting the findings of this study are available within the article and its Supplementary Information files and from the corresponding author upon reasonable request.
