# Supplementary material for: Structural basis for chemokine receptor CCR6 activation by the endogenous protein ligand CCL20
Source: Nat Commun. 2020 Jun 15;11:3031. doi: 10.1038/s41467-020-16820-6 (PMC7295996; doi:10.1038/s41467-020-16820-6)
Supplement: Supplementary file 1 — Supplementary Information [file 41467_2020_16820_MOESM1_ESM.pdf]

Supplementary Information

Structural basis for chemokine receptor CCR6 activation by the endogenous protein ligand CCL20

David Jonathan Wasilko[1], Zachary Lee Johnson[1], Mark Ammirati[1], Ye Che[1], Matthew C. Griffor[1], Seungil Han[1], Huixian Wu[1]*

[1] Discovery Sciences, Medicine Design, Pfizer Worldwide Research and Development, Groton, CT 06340

*To whom correspondence should be addressed: huixian.wu@pfizer.com

Sequences used in this study

Supplementary Figures 1-8

Supplementary Table 1

Sequences used in this study

Human CCR6

MKTIIALSYIFCLVFADYKDDDDAKLQTMGTADLEDNWETLNDNLKVIEKADNAAQVK
DALTKMRAAALDAQKATPPKLEDKSPDSPEMKDFRHGFDILVGQIDDALKLANEGKVK
EAQAAAEQLKTTRNAYIQKYLGSENLYFQGSGESMNFSDVFDSSEDYFVSVNTSYYSVD
SEMLLCSLQEVRQFSRLFVPIAYSLICVFGLLGNILVVITFAFYKKARSMTDVYLLNMAIA
DILFVLTLPFWAVSHATGAWVFSNATCKLLKGIYAINFNCGMLLLTCISMDRYIAIVQAT
KSFRLRSRTLPRSKIICLVVWGLSVIISSSTFVFNQKYNTQGSDVCEPKYQTVSEPIRWKL
LMLGLELLFGFFIPLMFMIFCYTFIVKTLVQAQNSKRHKAIRVIIAVVLVFLACQIPHNMV
LLVTAANLGKMNRSCQSEKLIGYTKTVTEVLAFLHCCLNPVLYAFIGQKFRNYFLKILK
DLWCVRRKYKSSGFSCAGRYSENISRQTSETADNDNASSFTMHHHHHHHHHH

Human CCL20

MADQLTEEQIAEFKEAFSLFDKDGDGTITTKELGTVMRSLGQNPTEAELQDMINEVDAD
GNGTIDFPEFLTMMARKMKDTDSEEEIREAFRVFDKDGNGYISAAELRHVMTNLGEKLT
DEEVDEMIREADIDGDGQVNYEEFVQMMTAKGRGS**ENLYFQA**SNFDCCLGYTDRILHP
KFIVGFTRQLANEGCDINAIIFHTKKKLSVCANPKQTWVKYIVRLLSKKVKNM

TEV site labelled in bold. Chemokine was cleaved prior to complex formation.

miniG$_{\alpha o}$

MGHHHHHHENLYFQGTLSAEERAALERSKAIEKNLKEDGISAAKDVKLLLLGADNSGK
STIVKQMKIIHGGSGGSGGTTGIVETHFTFKNLHFRLFDVGGQRSERKKWIHCFEDVTAII
FCVDLSDYNRMHESLMDFDSICNNKFFIDTSIILFLNKKDLFGEKIKKSPLTICFPEYTGPN
TYEDAAAYIQAQFESKNRSPNKEIYCHMTCATDTNNAQVIFDAVTDIIIANNLRGCGLY

Rat G$_{\beta}$

MHHHHHHGSLLQSELDQLRQEAEQLKNQIRDARKACADATLSQITNNIDPVGRIQMRTR
RTLRGHLAKIYAMHWGTDSRLLVSASQDGKLIIWDSYTTNKVHAIPLRSSWVMTCAYA
PSGNYVACGGLDNICSIYNLKTREGNVRVSRELAGHTGYLSCCRFLDDNQIVTSSGDTT
CALWDIETGQQTTTFTGHTGDVMSLSLAPDTRLFVSGACDASAKLWDVREGMCRQTFT
GHESDINAICFFPNGNAFATGSDDATCRLFDLRADQELMTYSHDNIICGITSVSFSKSGRL
LLAGYDDFNCNVWDALKADRAGVLAGHDNRVSCLGVTDDGMAVATGSWDSFLKIWN

Rat G$_{\gamma}$

MASNNTASIAQARKLVEQLKMEANIDRIKVSKAAADLMAYCEAHAKEDPLLTPVPASE
NPFREKKFFC

scFv16

METDTLLLWVLLLWVPGSTGDVQLVESGGGLVQPGGSRKLSCSASGFAFSSFGMHWV
RQAPEKGLEWVAYISSGSGTIYYADTVKGRFTISRDDPKNTLFLQMTSLRSEDTAMYYC
VRSIYYYGSSPFDFWGQGTTLTVSSGGGGSGGGGSGGGGSDIVMTQATSSVPVTPGESV

SISCRSSKSLLHSNGNTYLYWFLQRPGQSPQLLIYRMSNLASGVPDRFSGSGSGTAFTLTI
SRLEAEDVGVYYCMQHLEYPLTFGAGTKLELKAAALEVLFQGPHHHHHHHH

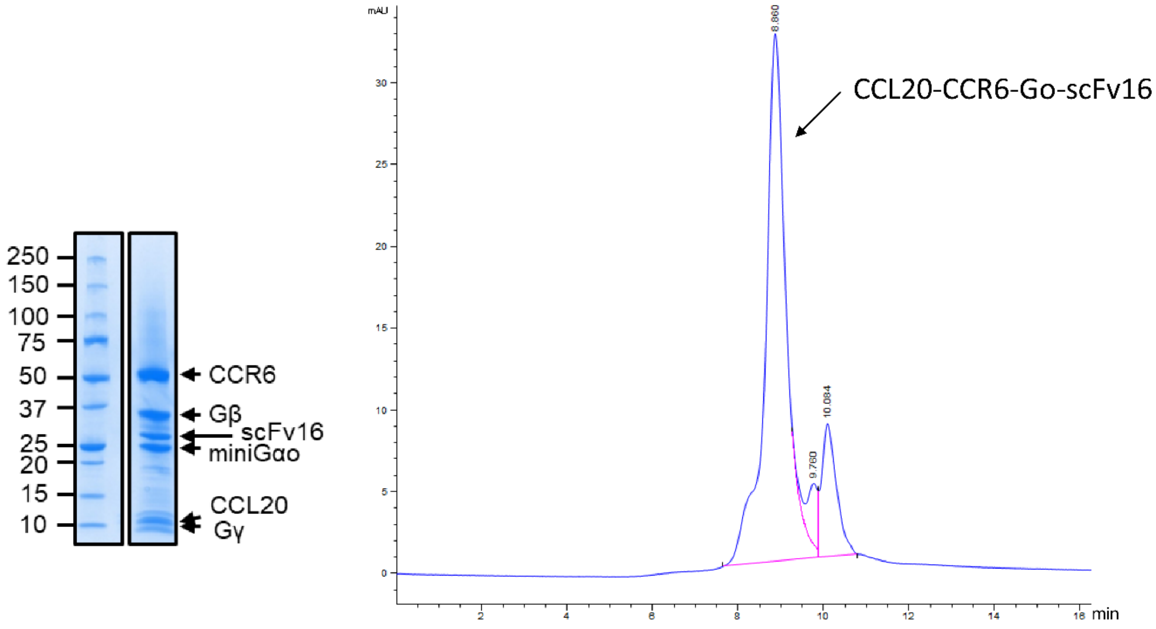

Supplementary Figure 1. Purification of the CCR6/CCL20-Go-scFv16 complex. Representative SDS-PAGE and size exclusion chromatography profile of the N-terminal BRIL-fused full-length human CCR6 in complex with CCL20, heterotrimeric Go, and scFv16.

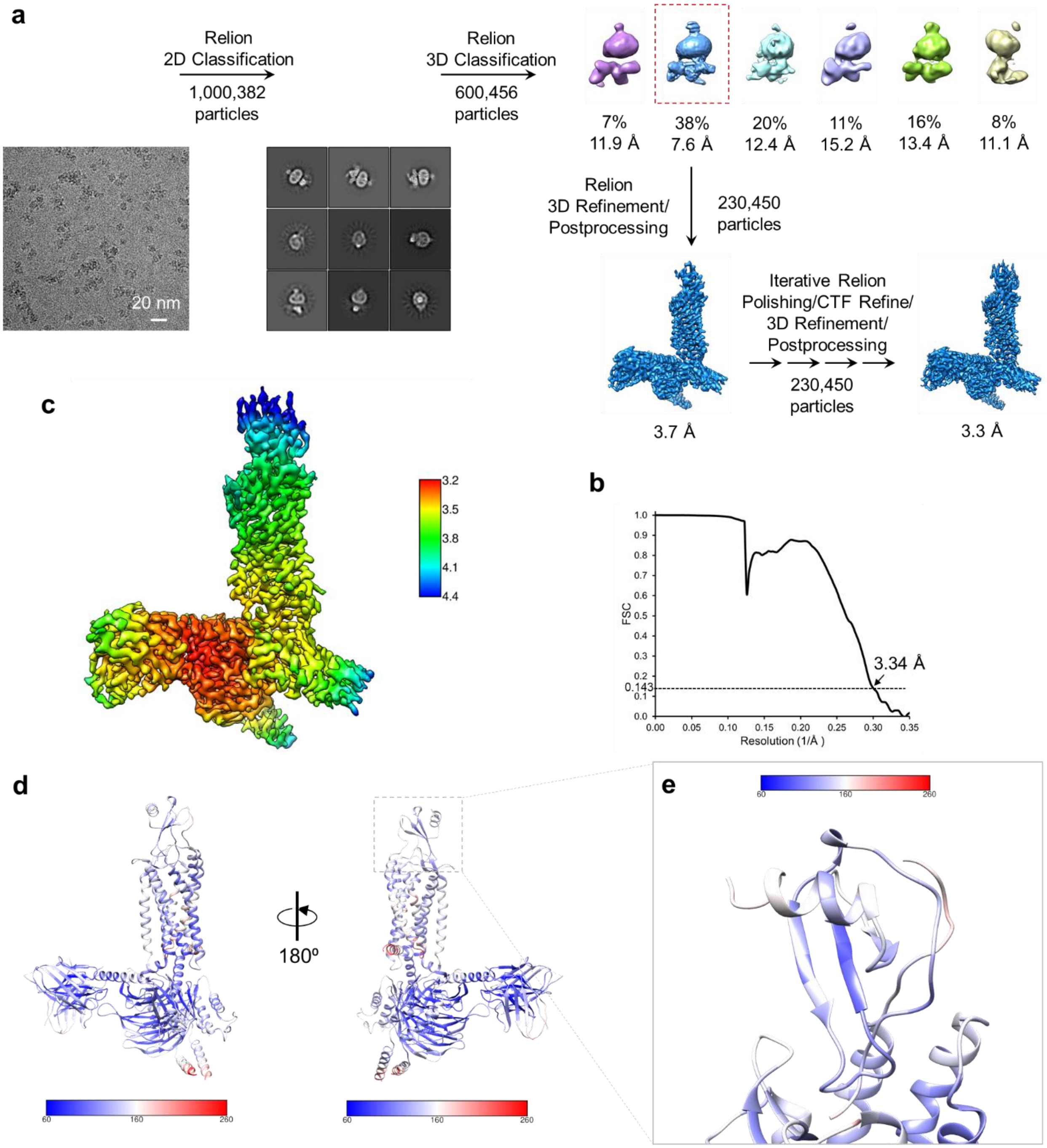

Supplementary Figure 2. Cryo-EM data processing. a, Flow chart of cryo-EM data processing, including a representative micrograph of the CCR6/CCL20-Go-scFv16 complex and representative 2D class averages showing distinct views and features of the complex. **b**, Fourier shell correlation (FSC) curve indicates an overall nominal resolution of 3.3 Å using the Gold-standard FSC=0.143 criterion. **c**, Density map coloured by local resolution. **d** and **e**, B-factor distributions of (**d**) the overall CCR6/CCL20-Go-scFv16 complex structure and (**e**) CCL20 and the chemokine-chemokine receptor binding interface.

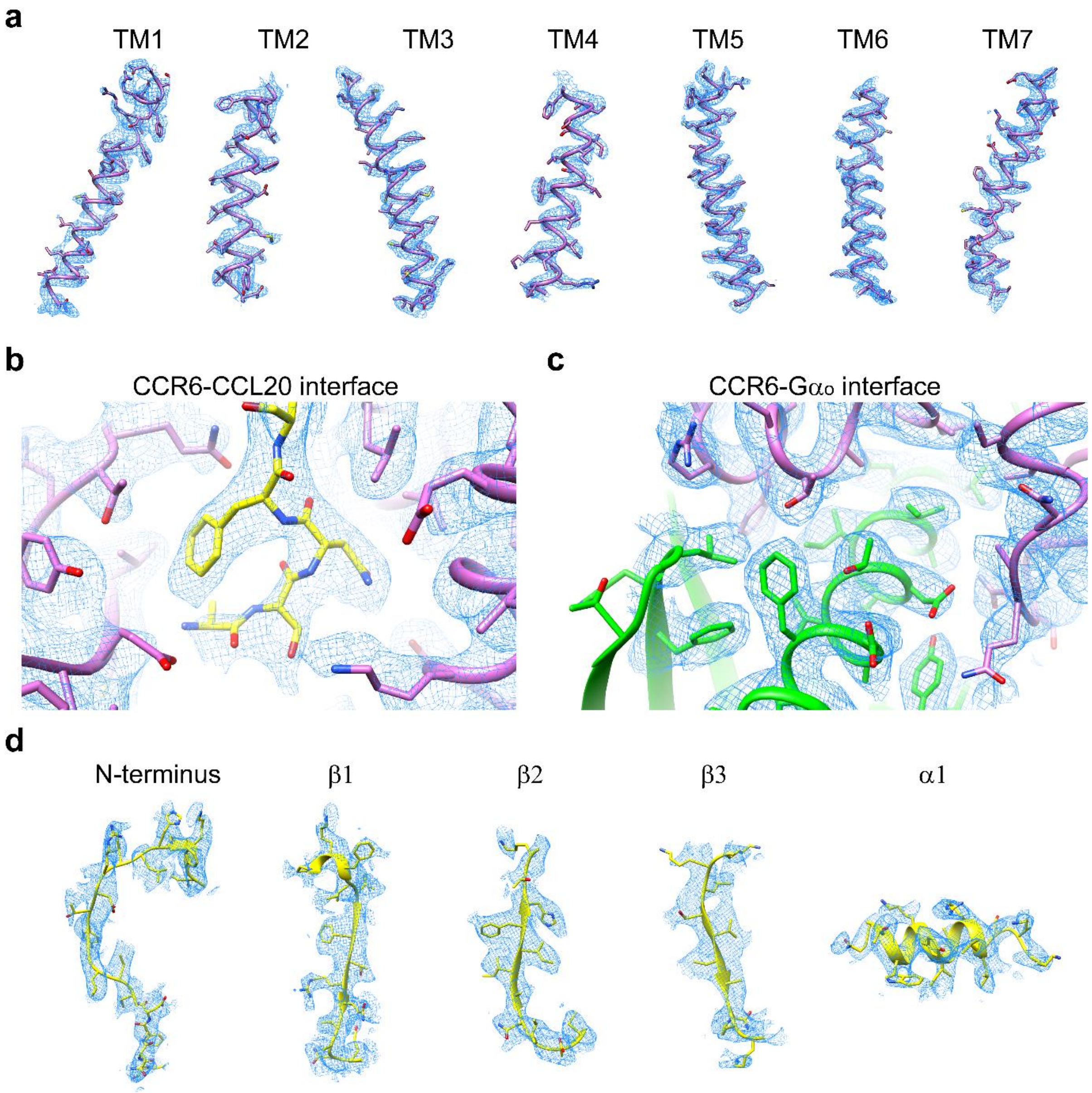

Supplementary Figure 3. Cryo-EM map quality. **a**, Density and model of the CCR6 transmembrane helices. **b**, Map and model of the CCL20 N-terminus-CCR6 interaction interface. **c**, Map and model of the α5 helix of the Gαo-CCR6 interaction interface. **d**, Map and model of the N-terminus (residues 1-16), β1 (residues 17-30), β2 (residues 31-42), β3 (residues 43-52), and α1 (residues 53-65) of CCL20.

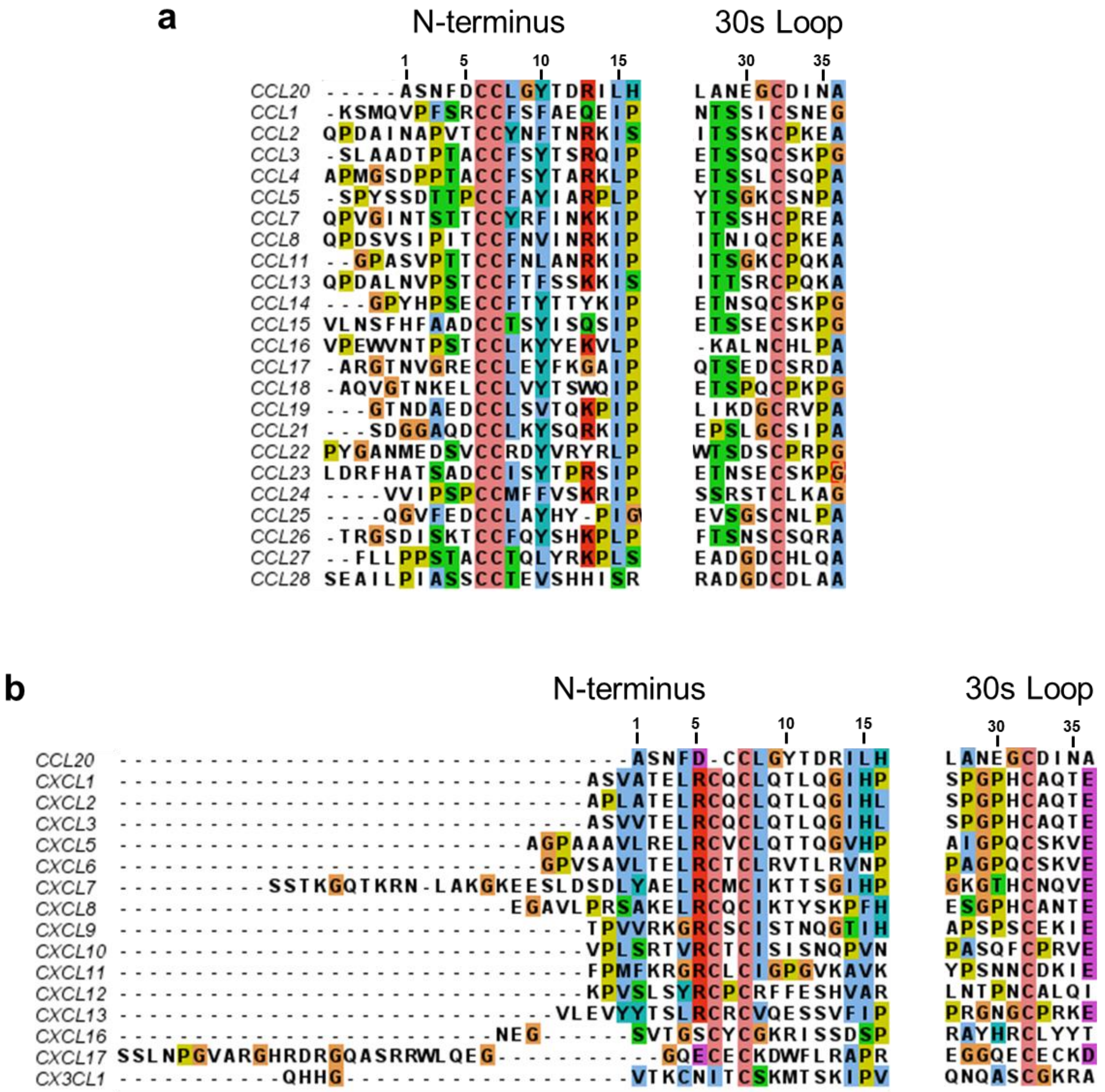

Supplementary Figure 4. Partial sequence (N-terminus and 30s loop) alignment of human chemokine agonists. a, Comparison of CCL20 and other CC chemokines. **b**, Comparison of CCL20 with human CXC and CX_3C chemokines.

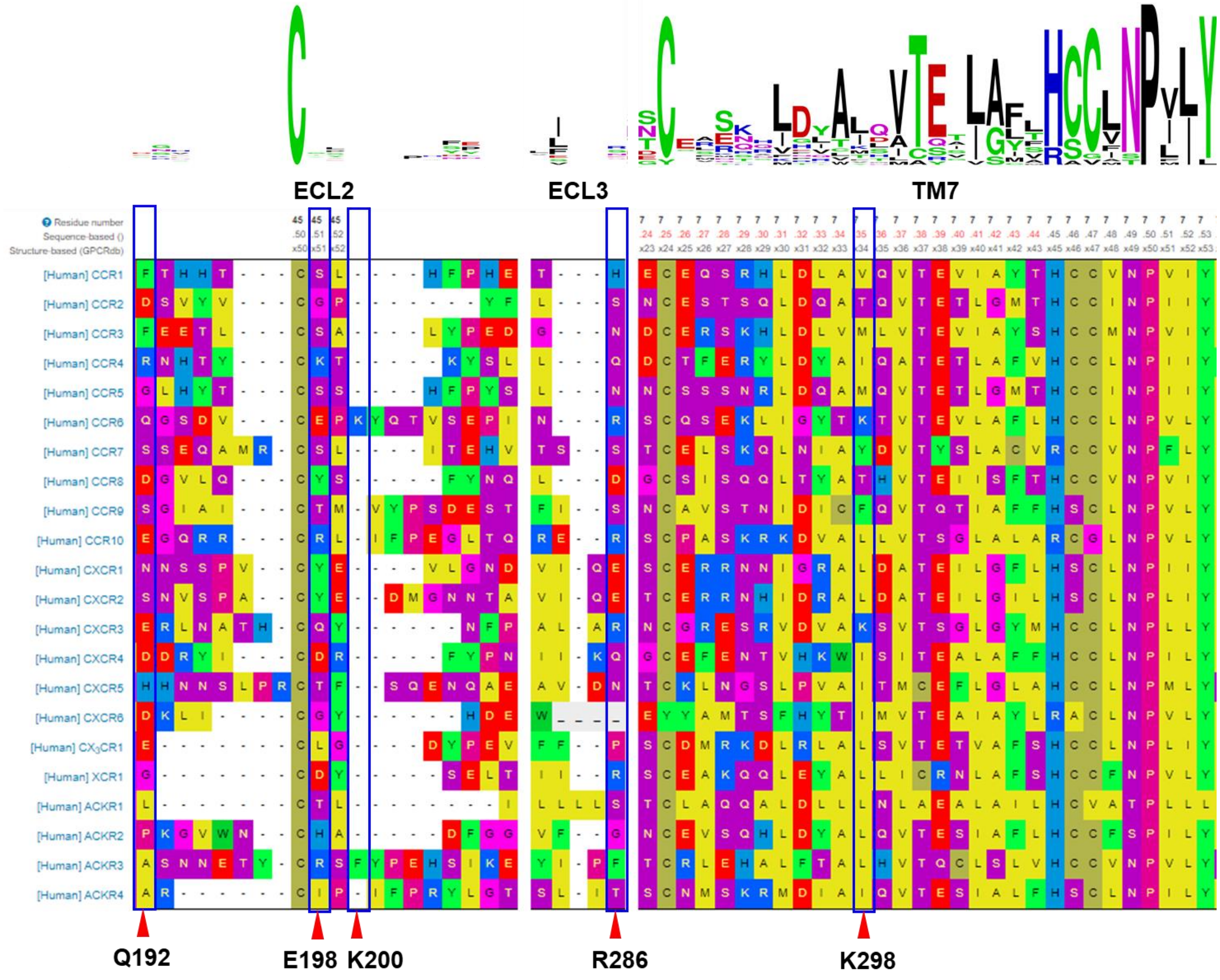

Supplementary Figure 5. Structure-based sequence alignment of ECL2, ECL3, and TM7 of human chemokine receptors. The CCR6 polar side-chains, Q192^{ECL2}, E198$^{45.51}$, K200^{ECL2}, R286^{ECL3}, and K298$^{7.35}$ that contribute to CCL20-specific recognition and their counterparts in other chemokine receptors are highlighted by boxes and red arrows.

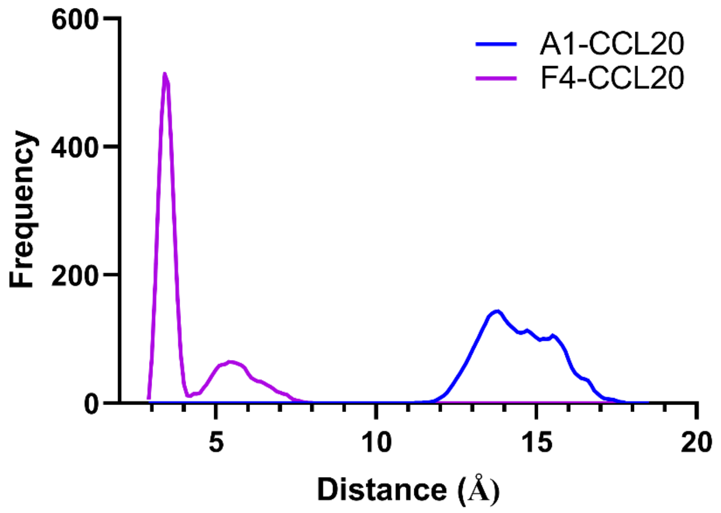

Supplementary Figure 6. Molecular dynamics simulation of CCR6 intramolecular salt bridge formation. Frequency distribution of the distance between CCR6 E198 and K298 side chains during molecular dynamics simulations using wild-type CCL20 (blue trace) and a truncated variant CCL20 (4-70) (purple trace).

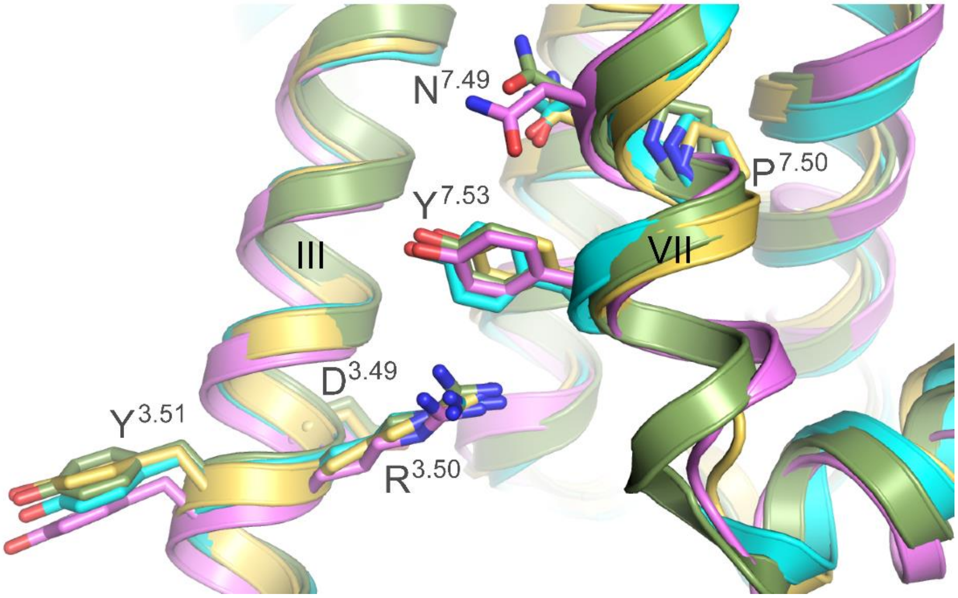

Supplementary Figure 7. Structural comparison of CCR6 and representative active GPCR-G protein complexes. The conserved motifs $D^{3.49}R^{3.50}Y^{3.51}$ and $N^{7.49}P^{7.50}xxY^{7.53}$ are shown in stick representation. The structures used in this comparison are: CCR6 (light magenta), β2AR-Gs (3SN6, yellow orange), 5HT1B-Go (6G79, dark green), and M1-G11 (6OIJ, cyan).

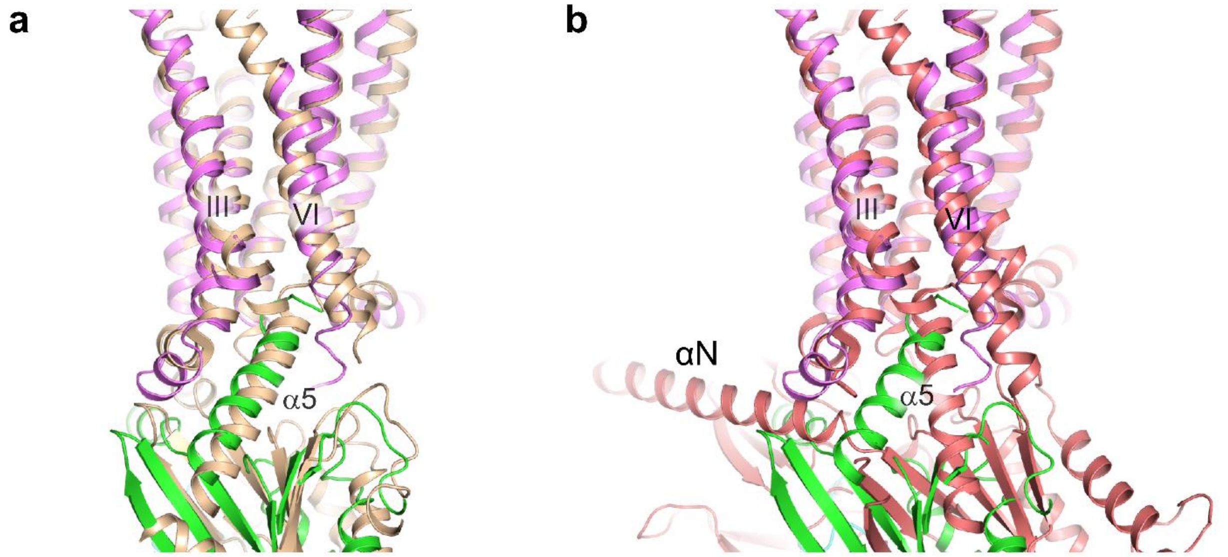

Supplementary Figure 8. CCR6-Go adopts the canonical state. a and **b**, Comparison of CCR6-Go (light magenta and green) with (**a**) the canonical state of NTS1R-Gi complex (6OS9, wheat) and (**b**) the non-canonical state of NTS1R-Gi (6OSA, deep salmon).

Supplementary Table 1. Cryo-EM data collection, refinement and validation statistics

	CCR6/CCL20-miniG$_o$-scFv16 Complex (EMD-21950) (PDB ID 6WWZ)
**Data collection and processing**	
Magnification	165,000x
Voltage (kV)	300
Electron exposure (e–/Å^2)	81.0
Defocus range (μm)	-0.6 to -2.0
Pixel size (Å)	0.87
Symmetry imposed	C1
Initial particle images (no.)	1,000,382
Final particle images (no.)	230,450
Map resolution (Å)	3.34
FSC threshold	0.143
**Refinement**	
Initial model used (PDB code)	6G79, 1M8A, 6DDE
Model resolution (Å)	3.34
FSC threshold	0.5
Map sharpening *B* factor (Å^2)	-76
Model composition	
Non-hydrogen atoms	9450
Protein residues	1212
B factors (Å^2)	
Protein	123.52
R.m.s. deviations	
Bond lengths (Å)	0.0143
Bond angles (°)	1.74
Validation	
MolProbity score	1.28
Clashscore	2.02
Rotamer outliers (%)	0.10
Ramachandran plot	
Favored (%)	95.56
Allowed (%)	4.44
Disallowed (%)	0.00
