# Supplementary material for: Structural basis for chemokine receptor CCR6 activation by the endogenous protein ligand CCL20
Source: Nat Commun. 2020 Jun 15;11:3031. doi: 10.1038/s41467-020-16820-6 (PMC7295996; doi:10.1038/s41467-020-16820-6)
Supplement: Supplementary file 2 — Reporting Summary [file 41467_2020_16820_MOESM2_ESM.pdf]

nature research

Corresponding author(s): Huixian Wu

Last updated by author(s): May 10, 2020

Reporting Summary

Nature Research wishes to improve the reproducibility of the work that we publish. This form provides structure for consistency and transparency in reporting. For further information on Nature Research policies, see Authors & Referees and the Editorial Policy Checklist.

Statistics

For all statistical analyses, confirm that the following items are present in the figure legend, table legend, main text, or Methods section.

n/a	Confirmed	
☐	☒	The exact sample size (*n*) for each experimental group/condition, given as a discrete number and unit of measurement
☐	☒	A statement on whether measurements were taken from distinct samples or whether the same sample was measured repeatedly
☒	☐	The statistical test(s) used AND whether they are one- or two-sided<br>*Only common tests should be described solely by name; describe more complex techniques in the Methods section.*
☒	☐	A description of all covariates tested
☒	☐	A description of any assumptions or corrections, such as tests of normality and adjustment for multiple comparisons
☒	☐	A full description of the statistical parameters including central tendency (e.g. means) or other basic estimates (e.g. regression coefficient) AND variation (e.g. standard deviation) or associated estimates of uncertainty (e.g. confidence intervals)
☒	☐	For null hypothesis testing, the test statistic (e.g. *F*, *t*, *r*) with confidence intervals, effect sizes, degrees of freedom and *P* value noted<br>*Give P values as exact values whenever suitable.*
☒	☐	For Bayesian analysis, information on the choice of priors and Markov chain Monte Carlo settings
☒	☐	For hierarchical and complex designs, identification of the appropriate level for tests and full reporting of outcomes
☒	☐	Estimates of effect sizes (e.g. Cohen's *d*, Pearson's *r*), indicating how they were calculated

Our web collection on statistics for biologists contains articles on many of the points above.

Software and code

Policy information about availability of computer code

Data collection	Automated data collection on the Titan Krios was performed using serialEM 3.8.0 beta
Data analysis	The following software was used in this study: MotionCor2-v1.3.0, CTFFIND-4.1, RELION-v3.0.5, ResMap-v1.9, UCSF Chimera-v1.14, Coot-v0.8.9, Phenix.real_space_refine (Phenix dev-3026), Refmac5 (CCP4 7.0), Molprobity-v4.4, Maestro 12.1.013 (Desmond Release 2019-3), GraphPad Prism-v8.0.2, PyMol-v2.2.0

For manuscripts utilizing custom algorithms or software that are central to the research but not yet described in published literature, software must be made available to editors/reviewers. We strongly encourage code deposition in a community repository (e.g. GitHub). See the Nature Research guidelines for submitting code & software for further information.

Data

Policy information about availability of data

All manuscripts must include a data availability statement. This statement should provide the following information, where applicable:

- Accession codes, unique identifiers, or web links for publicly available datasets
- A list of figures that have associated raw data
- A description of any restrictions on data availability

All data generated or analyzed during this study are included in this published article. Sequences of constructs used in this study are listed in Supplementary Figure 1. The cryo-EM density maps for the CCR6/CCL20-Go-scFv16 has been deposited in the Electron Microscopy Data Bank under accession code EMD-21950. The coordinates for the models of CCR6/CCL20-Go-scFv16 has been deposited in the Worldwide Protein Data Bank under accession code 6WWZ.

Field-specific reporting

Please select the one below that is the best fit for your research. If you are not sure, read the appropriate sections before making your selection.

☒ Life sciences ☐ Behavioural & social sciences ☐ Ecological, evolutionary & environmental sciences

For a reference copy of the document with all sections, see nature.com/documents/nr-reporting-summary-flat.pdf

Life sciences study design

All studies must disclose on these points even when the disclosure is negative.

Sample size	Cryo-EM particle sample size was not pre-determined by any statistical metrics. This sample size was limited by time allocation on microscopes. Subsequent data processing yielded a 3.34Å resolution final map, suggesting images we collected has sufficient number of useful particles to generate high-resolution structure.
Data exclusions	No data was systematically excluded. Over the course of refinement of our maps, particles with low signal, or particles that did not align well to the consensus map as determined by pre-established software criteria were excluded from final map calculations as implemented in RELION 3.0.5.
Replication	No replication studies were attempted, nor were necessary. Our primary data is a cryo-EM structure that was calculated according to standard procedures and does not need replicates.
Randomization	No randomization was necessary for this study. Our primary data is a calculated cryo-EM structure that was calculated according to standard procedures with freely available software and does not need randomization.
Blinding	No blinding was used nor necessary during data collection or analysis. As above, Our primary data is a calculated cryo-EM structure that was calculated according to standard procedures with freely available software and did not require blinding. We calculated our initial model abinitio to avoid bringing model bias into our map calculation, and in a sense, blinded the software.

Reporting for specific materials, systems and methods

We require information from authors about some types of materials, experimental systems and methods used in many studies. Here, indicate whether each material, system or method listed is relevant to your study. If you are not sure if a list item applies to your research, read the appropriate section before selecting a response.

Materials & experimental systems

n/a	Involved in the study	
☐	☒	Antibodies
☐	☒	Eukaryotic cell lines
☒	☐	Palaeontology
☒	☐	Animals and other organisms
☒	☐	Human research participants
☒	☐	Clinical data

Methods

n/a	Involved in the study	
☒	☐	ChIP-seq
☒	☐	Flow cytometry
☒	☐	MRI-based neuroimaging

Antibodies

Antibodies used	scFv16 was an antibody that binds the Gi/o heterotrimer. Its sequence was acquired from the published papers https://doi.org/10.1038/s41586-018-0219-7 and https://doi.org/10.1038/s41586-018-0241-9.<br>Anti-FLAG M2 affinity gel was also used in this study (Millipore Sigma, catalog #A2220)
Validation	scFV16 has been validated as binders to Gi/o heterotrimer by the published papers https://doi.org/10.1038/s41586-018-0219-7 and https://doi.org/10.1038/s41586-018-0241-9. Binding was again confirmed by size-exclusion chromatography on purified protein, and the subsequent cryo-electron microscopy data collection. scFv16 could be built into density map and register was confirmed.<br>The binding of Anti-FLAG M2 affinity gel was validated by the manufacturer: https://www.sigmaaldrich.com/Graphics/COfAInfo/SigmaSAPQM/COFA/A2/A2220/A2220-BULK________SLCF5876__.pdf

Eukaryotic cell lines

Policy information about cell lines

Cell line source(s)	Sf9, ATCC; Origami 2, Novagen.

Authentication	Cell lines are maintained by the supplier. No additional authentication was performed by the authors of this study.
Mycoplasma contamination	Cell lines are tested by manufacturer for contamination, but were not further tested by the authors of this study.
Commonly misidentified lines (See ICLAC register)	None used.
